# Ferroelectric Metal in Tetragonal BiCoO_3_/BiFeO_3_ Bilayers and Its Electric Field Effect

**DOI:** 10.1038/srep20591

**Published:** 2016-02-03

**Authors:** Li Yin, Wenbo Mi, Xiaocha Wang

**Affiliations:** 1 Tianjin Key Laboratory of Low Dimensional Materials Physics and Preparation Technology, Faculty of Science, Tianjin University, Tianjin, 300072, China; 2 Tianjin Key Laboratory of Film Electronic & Communicate Devices, School of Electronics Information Engineering, Tianjin University of Technology, Tianjin, 300384, China

## Abstract

By first-principles calculations we investigate the electronic structure of tetragonal BiCoO_3_/BiFeO_3_ bilayers with different terminations. The multiferroic insulator BiCoO_3_ and BiFeO_3_ transform into metal in all of three models. Particularly, energetically favored model CoO_2_-BiO exhibits ferroelectric metallic properties, and external electric field enhances the ferroelectric displacements significantly. The metallic character is mainly associated to *e*_*g*_ electrons, while *t*_*2g*_ electrons are responsible for ferroelectric properties. Moreover, the strong hybridization between *e*_*g*_ and O *p* electrons around Fermi level provides conditions to the coexistence of ferroelectric and metallic properties. These special behaviors of electrons are influenced by the interfacial electronic reconstruction with formed Bi-O electrovalent bond, which breaks O_A_-Fe/Co-O_B_ coupling partially. Besides, the external electric field reverses spin polarization of Fe/Co ions efficiently, even reaching 100%.

Multiferroics with ferroelectricity, ferromagnetism or ferroelasticity simultaneously have great potential applications in information storage, electronic devices and sensors^1^,^2^,^3^. Particularly, magneto-electric multiferroics, where the spontaneous ferroelectric polarization can be controlled by an external magnetic field or vice versa, are found in perovskite-type transition metal oxides providing bright prospect for novel spintronic devices^4^,^5^,^6^,^7^,^8^. Oxide heterostructures exhibit unique properties absent in the corresponding isolated parent compounds, therefore it is an effective means to study emergent physics of correlated electrons, such as, metal-insulator transition^9^, two-dimensional electron gas and sharp interfaces at LaAlO_3_/SrTiO_3_ interfaces^10^,^11^,^12^,^13^, orientation-dependent magnetism and so on^14^. Besides, recent technology advances in oxide synthesis at the atomic level make artificially designing heterostructures feasible^15^. We attempt to combine two perovskite-like multiferroics into bilayers aimed at inducing novel electronic and magnetic states, providing theoretical support for new multifunction devices as well. We pay attention upon Bi-based perovskite materials, whose ferroelectric properties originates from a lone pair of (6s)^2^ electrons^16^,^17^,^18^, and select tetragonal BiFeO_3_ (BFO) and BiCoO_3_ (BCO) as multiferroics candidates.

The perovskite BFO is the only known room-temperature single-phase magneto-electric multiferroic material, which is intensively studied in the last decade, with a high ferroelectric Curie temperature of 1103 K and antiferromagnetic Neel temperature of 643 K^19^,^20^,^21^,^22^,^23^, exhibiting weak magnetism at room temperature due to a residual moment from a canted spin structure^24^. Notablely, tetragonal BFO has much higher spontaneous polarization of 150 μC/cm^2^ and charge transfer excitations than rhombohedral phase, and gets considerable high resistance changes in ferroelectric tunnel junctions^25^,^26^,^27^,^28^,^29^. The resistance changes in ferroelectric tunnel junctions based on tetragonal BFO are considerably high (OFF/ON ratio >10000) among known ferroelectric tunnel junctions^30^. BCO has been suggested to be a promising multiferroic material, which is predicted to exhibit a giant polarization and extremely high transition temperature^31^,^32^. The ferroelectricity of BCO is found to be primarily driven by the lone-pair activity of Bi^3+^, and magnetism being driven by the high-spin state of Co^3+^ in a C-type antiferromagnetic structure below a Neel temperature of 420 K^33^,^34^. And, BFO and BCO with large spontaneous ferroelectric polarization have great potential application in electrically controllable devices^35^,^36^,^37^,^38^,^39^. Besides, compounding BFO with BCO is accessible experimentally in the form of epitaxial thin film^40^ and the BFO/BCO multiferroic solid solutions are studied theoretically^41^. Previous studies show that the antiferromagnetic insulator BiFeO_3_ can exhibit ferromagnetism in BiFeO_3_/La_2/3_Sr_1/3_MnO_3_ interface^42^,^43^ and two-dimensional electron gas in BiFeO_3_/SrTiO_3_ interface^44^, demonstrating that heterointerface is significant in BiFeO_3_-based bilayers. However, the heterostructures by constructing BiFeO_3_ with another multiferroic BiCoO_3_ may present some fantastic properties based on its multiferroic characteristics.

In this paper, we study the electronic structure of BCO/BFO bilayers with different terminations and investigate the external electric field effect on the bilayers by first-principles calculations. We find that energetically favored model CoO_2_-BiO exhibits ferroelectric metallic properties due to the division of *e*_*g*_ and *t*_*2g*_ electrons as well as *e*_*g*_-*p* hybridization. Additional, external electric field enhances the ferroelectric displacements markedly. These special behaviors of electrons are influenced by the interfacial electronic reconstruction with formed Bi-O electrovalent bond, which breaks O_A_-Fe/Co-O_B_ coupling partially. Our results indicate that interfacial coupling and electric field play key roles on the novel ferroelectric metallic properties of model CoO_2_-BiO, which provides opportunities for developing functional nanoelectronic devices.

## Calculation Details

Our first-principle calculations are performed using density functional theory (DFT) within the local spin-density approximation (LSDA), based on the projector augment wave (PAW) pseudo-potentials. The energy cutoff for plane wave basis set is 500 eV and the Brillouin zone is sampled with Γ-centered 5 × 5 × 5 and 5 × 5 × 1 *k* point meshes for bulk compounds and bilayers respectively, providing numerical convergence of 10^−5 ^eV. All the structures are fully relaxed until the maximum Hellmann-Feynman forces on each atom are less than 0.02 eV/Å. Aimed at getting reasonable results, we include an on-site Coulomb repulsion of *U* = 6 eV for Co 3*d* states^45^, and *U* = 4.5 eV for Fe 3*d* states^46^,^47^, which are sufficient to describe the related bulk properties.

**Figure 1:**
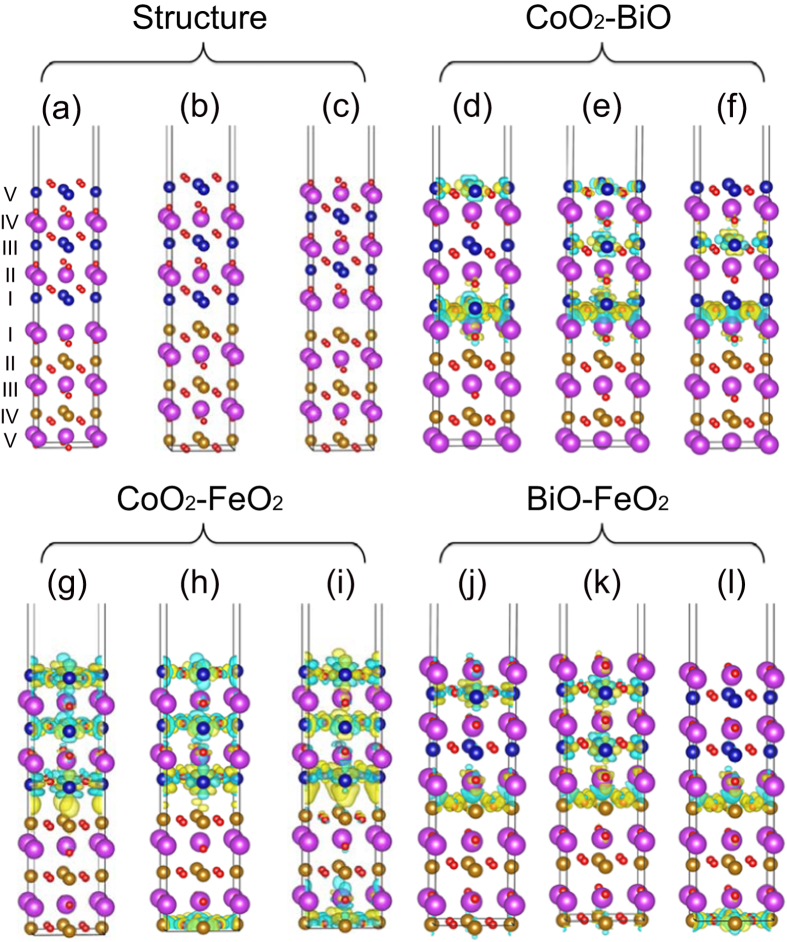
(**a–c**) side views of BCO/BFO bilayers for model CoO_2_-BiO, CoO_2_-FeO_2_ and BiO-FeO_2_ respectively. (**d**–**f**) charge density difference of model CoO_2_-BiO (isosurface value 0.008 e/Å^3^) in *E* = 0, 6 and 10 mV/Å respectively, (**g–i**) for model CoO_2_-FeO_2_ (isosurface value 0.003 e/Å^3^)and (**j–l**) for model BiO-FeO_2_ (isosurface value 0.008 e/Å^3^). The red spheres stand for O, dark yellow for Fe, dark blue for Co and purple for Bi. The yellow and blue isosurfaces represent accumulation and depletion of electrons, respectively.

Tetragonal phase of the multiferroic BFO used in this work has a perovskite-type structure with a lattice constant of *a* = 3.770 Å and *c/a* = 1.233 in space group *P4 mm*^47^. The primitive cell of tetragonal BFO contains one molecule with one Bi atom located at (0.0, 0.0, 0.0), one Fe atom at (0.5, 0.5, 0.439), one axial O_A_ atom at (0.5, 0.5, −0.170) in BiO layer and two equatorial O_B_ atoms at (0.0, 0.5, 0.294) and (0.5, 0.0, 0.294) in FeO_2_ layer. The magnetic character of BFO is G-type where the Fe atoms are coupled ferromagnetically within the (111) planes and antiferromagnetically between adjacent planes. Bulk BCO is an antiferromagnetic insulator of C-type which is the most stable magnetic order in BCO^48^, where Co ions are aligned antiferromagnetically in the *xy* plane and ferromagnetically along the *z* axis, with a lattice constant of *a* = 3.729 Å and *c/a* = 1.267 in space group *P4 mm*^45^. The used experiment value of atomic coordinates in BCO are Bi (0.0, 0.0, 0.0), Co (0.5, 0.5, 0.5669), one axial O_A_ (0.5, 0.5, 0.2034) and two equatorial O_B_ (0.5, 0.0, 0.73)^33^. Bi ions locate in the corner sites, yet Fe (or Co) ions and O ions which ought to occupy the body and face centered sites respectively move from center sites in the *z* direction owing to ferroelectric spontaneous polarization. In the supercells of BCO/BFO studied here, a 28 Å vacuum space in *z* direction is used to separate the interaction between periodic images and the supercells are built by placing five BCO atomic layers on the top of five BFO atomic layers within *p*(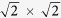) periodicity giving altering layers of Bi_2_O_2_ and Co_2_O_4_ (Fe_2_O_4_) along the [001] direction. In experiments, the thin films must present one surface that is exposed in the vacuum, even though the sample is a multilayered structure. Hence, the bilayer geometry with vacuum should be calculated. Meanwhile, the difference of optimized geometry and atomic position can affect the multiferroics of the sample. In superlattice structure, there are two interfaces which might influence the relaxation of the atoms, so that each atomic position should be different from the case of bilayer with vacuum. Meanwhile, in order to study the effect of external electric field, the bilayer with vacuum is necessary. We apply external electric field for the bilayers in *z* direction and switch on the potential correction mode. The calculated in-plane lattice mismatch between BFO(001) and BCO(001) is 1.1%, indicating a good lattice match. We set up three BCO/BFO bilayers with different terminations to investigate the interfacial properties, as shown in Fig. 1(a–c).

The stable pattern is determined by calculating the work of separation, i.e., the cohesive energy between BCO and BFO, 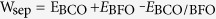, where *E*_*BCO/BFO*_ is the total energy of the bilayers, *E*_*BCO*_ and *E*_*BFO*_ represent the energies of the same supercell containing either the BCO or BFO parts (i.e., we keep the equilibrium structure obtained for the bilayers). For illustrating the nature of the charge transfer at BCO/BFO interface, we calculate the charge density difference by subtracting the charge densities of isolated BFO and BCO parts from the charge density of bilayers as shown in Fig. 1(d–l). The electronic structures of isolated BFO and BCO are calculated by freezing the atoms of the respective component at the supercell positions.

## Results and Discussion

**Figure 2:**
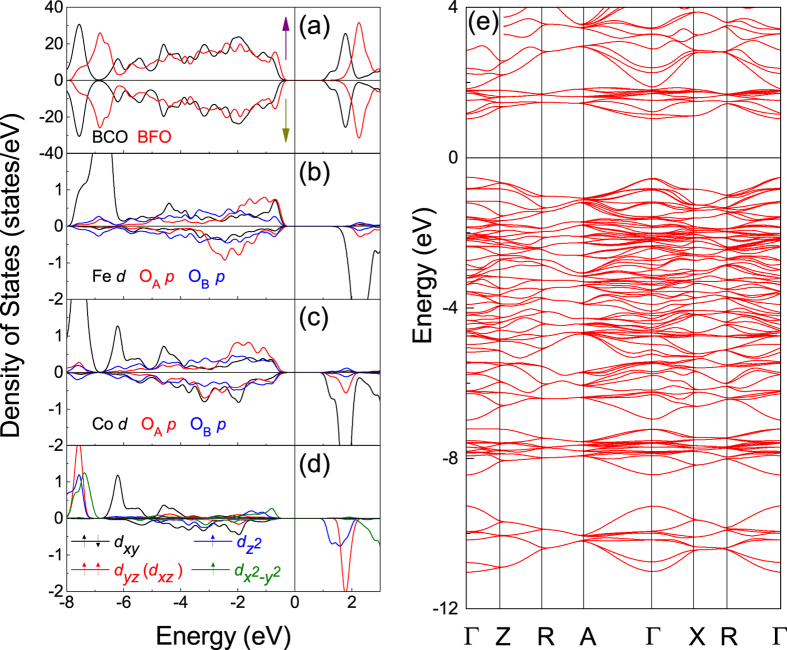
(**a**) Total DOS for bulk BFO and BCO. (**b**) Partial DOS for bulk BFO. (**c**) Partial DOS for bulk BCO. (**d**) DOS for Co *d* electrons in bulk BCO. The Fermi level is indicated by vertical lines and set to zero. (**e**) Spin-up band structure of bulk BCO.

First, we analyze the total and projected densities of states (DOS) of fully relaxed bulk BFO and BCO shown in Fig. 2. For BFO, the charge transfer gap is determined by the filled oxygen 2*p* band and the unoccupied 3*d* band of Fe, and the calculated band gap of 1.93 eV is in good agreement with previous calculations^49^, but inconsistent with the experimental value of 3.10 eV^50^, as a result of using the LSDA approximation. The Fe spins are antiparallel and the corresponding DOS is symmetrical, so we only show one. The calculated Fe magnetic moments are ±4.107 μ_B_ per atom. For BCO, the calculated total DOS is similar with previous calculations^45^, and the Co ions are in high-spin state which is consistent with the experimental result^33^, as shown in Fig. 2(a,d). The spin-up and spin-down band structures are completely compatible, so we only show spin-up structure in Fig. 2(e). We find that the strong correlated effect of Co 3*d* is well described with a band gap of 1.52 eV and the Co magnetic moments are ±3.035 μ_B_ per atom, which are in good agreement with the experimental values of 1.7 eV and 3.24 μ_B_^33^,^51^. These bulk results reveal that the used parameters in the present work are reasonable.

**Figure 3:**
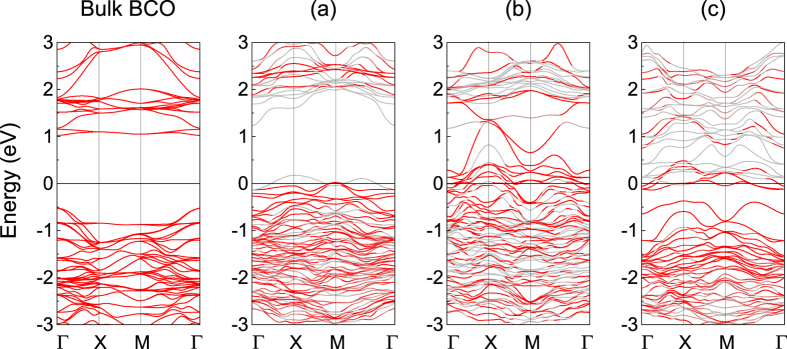
Spin-up band structure of bulk BCO, model (**a**) CoO_2_-BiO, (**b**) CoO_2_-FeO_2_ and (**c**) BiO-FeO_2_ bilayers. The red color indicates BCO and gray BFO. *E*_*F*_ = 0 eV.

We carry out the electronic band structures of three models and separate out the BCO’s contribution to demonstrate the changes of the electronic states in BCO by comparing with bulk BCO states in same path, as shown in Fig. 3. Obviously, both BCO and BFO transforms into metal in all of three interfacial models and BCO undergoes a dramatic change, revealing that interfacial compound probably is an efficient method to explore emergent physics as well. The strong interfacial effect is also reflected by the remarkable accumulation and depletion of electrons at interfaces, as shown in Fig. 1(d–l). Bi and O ions combine with each other in the form of electrovalent bond with Bi depleting and O accumulating electrons in the interfacial regions of model CoO_2_-BiO and BiO-FeO_2_, see Fig. 2(d,j). For model CoO_2_-FeO_2_, apparent accumulation of electrons between Co and Fe occurs at the interfacial regions revealing that Co and Fe ions combine via metallic bond we propose, as shown in Fig. 2(g). The calculated cohesive energies demonstrate that model CoO_2_-BiO is the most stable structure with a considerably large value of 11.474 eV and model CoO_2_-FeO_2_ is very unstable with a negative value, as listed in Table 1, which is reasonable since the interfaces in model CoO_2_-BiO and BiO-FeO_2_ are similar with the structures of bulk BCO and BFO, while model CoO_2_-FeO_2_ totally not.

**Figure 4:**
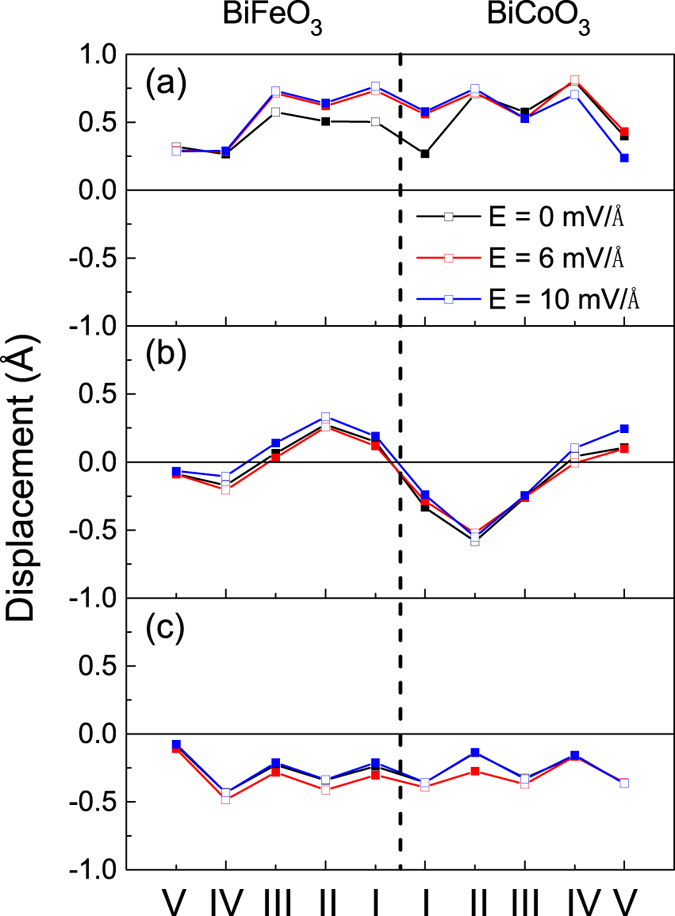
The change of Bi-O and B-O polar displacements in each layer along [001] direction in model (**a**) CoO_2_-BiO, (**b**) CoO_2_-FeO_2_ and (**c**) BiO-FeO_2_ due to different values of external electric field, B = Co/Fe. Open square symbols represent Bi-O displacements, and solid square symbols represent B-O displacements.

We further analyze the geometric structure of three models and the Bi-O, Fe-O and Co-O polar displacements of three models in one layer along [001] direction are calculated by subtracting the position of O ions. The black lines in Fig. 4 indicate that the displacements of model CoO_2_-BiO is larger than the other two models and we list the average values in Table 1. It is obvious that the displacements in model CoO_2_-BiO is almost 50% larger than model CoO_2_-FeO_2_ and model BiO-FeO_2_ and nearly three quarters of correspond bulks. Therefore, model CoO_2_-BiO exhibit metallic properties with remarkable ferroelectric structures since tetragonal BCO and BFO are typical displacive ferroelectrics originating from relative displacement of positive and negative ions^27^,^48^,^52^,^53^. To further investigate its ferroelectric properties, we add an electric field to all of three bilayers considering the strong electric field effect on ferroelectrics owing to the spontaneous polarization. Based on the experimental study on bulk^52^,^54^, we add the electric field of 6 and 10 mV/Å (i.e., 600 and 1000 kV/cm) respectively and calculated the relative displacements of positive and negative ions in same layer along *z* axis, as shown in Fig. 4. We find that the polarization displacements in model CoO_2_-FeO_2_ and model BiO-FeO_2_ with electric field (see red and blue lines) are close to the situation without electric field (see black lines) shown in Fig. 4(b,c), but the polarization shifts in model CoO_2_-BiO are enhanced greatly on the condition of applied electric field, particularly at the interfacial regions as shown in Fig. 4(a). This result further confirms the ferroelectric metallic properties of model CoO_2_-BiO and demonstrates that external electric field can modulate the ferroelectric polarization. Figure 1(d) reveals the strong interfacial coupling by Bi-O electrovalent bonds in the interfacial regions of model CoO_2_-BiO, and we further notice that the interfacial Bi-O bonds exist even in applied electric field, as shown in Fig. 1(e,f). This short-range pair interaction makes the ferroelectric polarization properties of bulks preserved in bilayers and lowers the electrostatic energy further stabilize the bilayers structure.

On the other hand, the structure of models CoO_2_-BiO and BiO-FeO_2_ contains two asymmetry surfaces and might be as polar as LaAlO_3_/SrTiO_3_ interface within a large “internal” electric field^9^,^55^, which automatically gives rise to the metallicity of the system. We check the same asymmetry geometry in pure BFO and BCO, which possesses the form (BiO-*M*O_2_)_*n*_ within 15 Å vacuum space in *z* direction (*M* = Fe/Co, n = 2, 3, 4). The calculated band structures indicate that such pure BFO is insulating when n = 2/3 but exhibits metallic in *n* = 4, while the pure BCO is metallic and not affected by *n*. Hence, the asymmetry structure is important for the metallic characters in CoO_2_-BiO model. Furthermore, such pure metallic properties in BFO and BCO are distinguished from the metallic characters in model CoO_2_-BiO. Firstly, both uppermost valence band (UVB) and lowest conduction band (LCB) approach the Fermi level in (BiO-FeO_2_)_4_, while the LCB of isolated BFO in model CoO_2_-BiO is far away from the Fermi level (see Fig. 3a). Secondly, the UVB in pure BCO overlaps with the Fermi level heavily, while the UVB of isolated BiCoO_3_ in CoO_2_-BiO model only approach the Fermi level (see Fig. 3a). It is obvious that the strong interfacial couplings have a great effect on the metallic characters in CoO_2_-BiO model.

**Figure 5:**
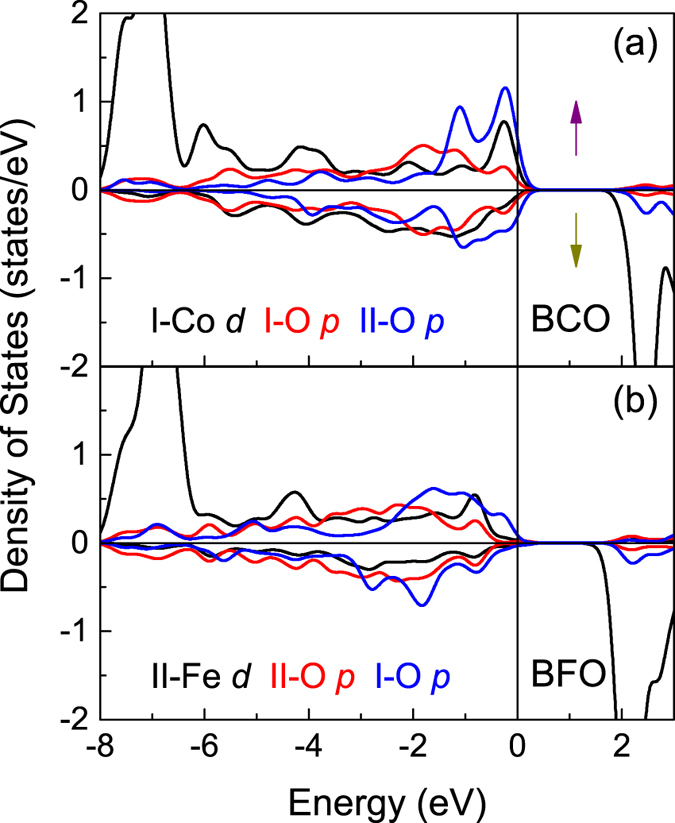
DOS for Fe, Co and O ions in interfacial regions of model CoO_2_-BiO. *E*_*F *_= 0 eV.

**Figure 6:**
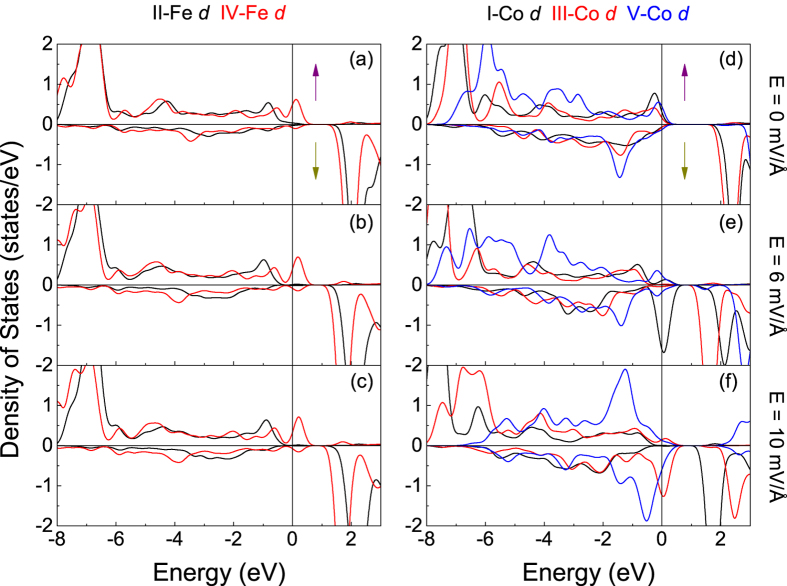
Partial DOS for (**a**–**c**) Fe and (**d**–**f**) Co in each layer of model CoO_2_-BiO on different values of electric field. *E*_*F *_= 0 eV.

Next, we analyze the electronic DOS distribution of ions in the interfacial regions of model CoO_2_-BiO in detail shown in Fig. 5. We find that, for BCO, I-Co *d* electrons hybridize with II-O *p* electrons distinctly but interact weakly with I-O *p* electrons in the energy range from −3 eV to Fermi level (*E*_*F*_) as indicated in Fig. 5(a). Similarly, for BFO, II-Fe *d* electrons hybridize obviously with II-O *p* electrons while lightly with I-O *p* electrons in the energy window from −1.2 to −0.3 eV as shown in Fig. 5(b). The label “I-Co” represents the Co ions in layer I as shown in Fig. 1(a), and this kind of definition is used in the whole letter. For bulk BCO and BFO, Fe/Co ions hybridize with O_A_ and O_B_, along with clear O alignment as shown in Fig. 2(b,c). For model CoO_2_-BiO, the interfacial Bi-O bonds make I-O *p* electrons in BFO and BCO change dramatically and break the balance of O_A_-Fe/Co-O_B_ partially, which further influences the metallic properties of bilayers with retained ferroelectric properties. Then, we analyze the Fe/Co DOS in each layer in different on the condition of electric field or not, as shown in Fig. 6. The Fe electronic distribution varies gently as indicated by Fig. 6(a–c), while Co ions change heavily shown in Fig. 6(d–f). We define spin polarization 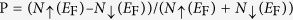 in terms of the total DOS in the spin-up *N*_↑_ and spin-down *N*_↓_ channels respectively, and find that the spin polarization of I-Co is reversed from 70% to −89% on the condition of *E* = 6 mV/Å by comparing Fig. 6(d) with Fig. 6(e). Besides, the spin polarization of III-Co and V-Co are reversed from 49% to −82% and 54% to −62% respectively on the condition of *E* = 10 mV/Å according to Fig. 6(d,f). The apparent positive-negative spin polarization reverse in Co ions demonstrates that electric field not only can be used to induce magnetic moments via magneto-electric effect as previous report^2^, but also can reverse spin polarization. The Fe/Co magnetic moments are listed in Table 2, which are influenced heavily by interfacial effect and electric field. The numbers of Fe/Co magnetic moments in same layer are equal but with different signs, so we only list the positive numbers in Table 2. In addition, the Co magnetic moments are changed easily, which is reasonable since the Co ions possess flexible possibilities of high, intermediate and low spin states. The electronic rearrangements of Co caused by interfacial coupling are also reflected by charge density difference since electrons with different orbital contours increase or decrease, shown in Fig. 1(d–l).

**Figure 7:**
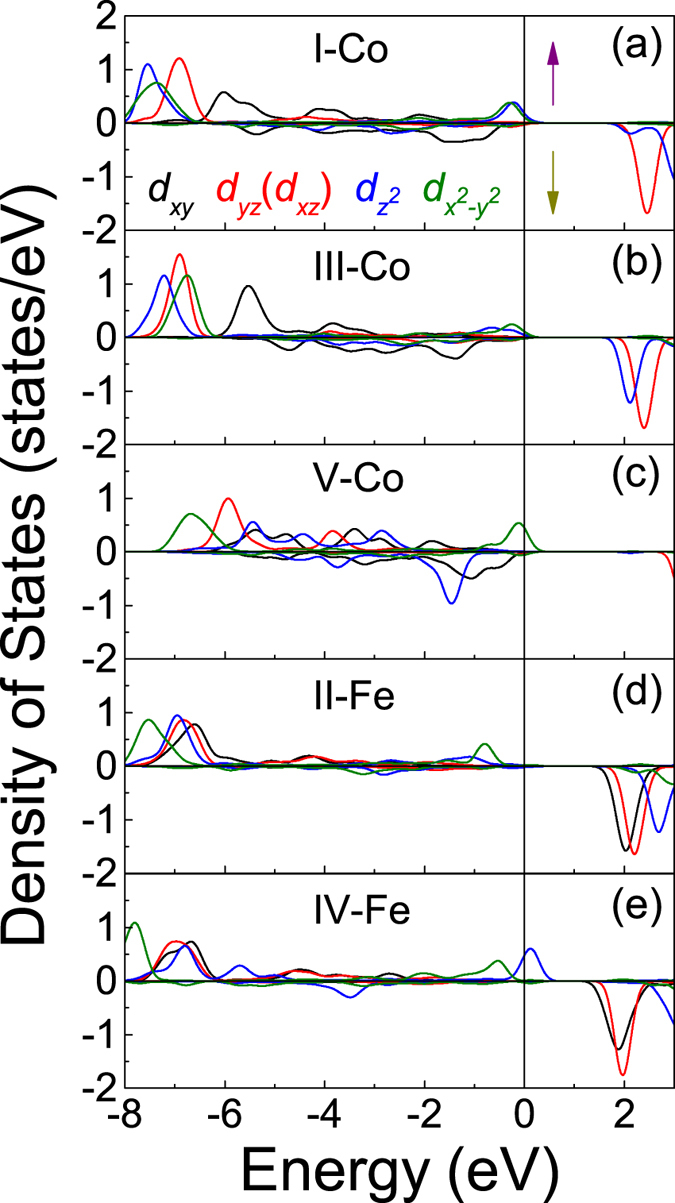
DOS for Co/Fe *d* electrons in each layer of model CoO_2_-BiO. *E*_*F*_ = 0 eV.

Although it is widely believed that metals cannot exhibit ferroelectricity since the static internal electric fields are screened by conduction electrons^56^, the ferroelectric metal is theoretically proposed by Anderson and Blount in 1965^57^. Recently LiOsO_3_ is identified as the first typical example^58^, and the microscopic mechanism for the ferroelectric-like structural transition in a metal are investigate widely^59^,^60^. The Mott multiferroic based on LiOsO_3_ is predicted by compounding with LiNbO_3_ as well^61^. However, in our model CoO_2_-BiO, the ferroelectrics are transformed into metal from insulator via interfacial coupling, which is opposite the LiOsO_3_-type metal into ferroelectric transition. The itinerant *d* electrons can screen the electric fields and inhibit the electrostatic forces, so we analyze the *d* electron states of Fe/Co ions in each layer of model CoO_2_-BiO on purpose as shown in Fig. 7. We find that the metallic property is associated to the electrons in *e*_*g*_ orbitals (i.e., 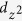 and 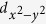), and these electrons hybridize with O *p* electrons around *E*_*F*_ according to Fig. 5. However, the electrons in *t*_*2g*_ orbitals (i.e., *d*_*xy*_, *d*_*xz*_, and *d*_*yz*_) have no contribution to the metallic character, which are responsible for the ferroelectric properties as shown in Figs 2(d) and 7. Therefore, although the specific *e*_*g*_ electrons exhibit metallic property, they simultaneously hybridize with O *p* electrons, which makes ferroelectric and metallic features coexist. And we argue that this special electron occupation is tightly associated with the interfacial coupling as mentioned above.

**Figure 8:**
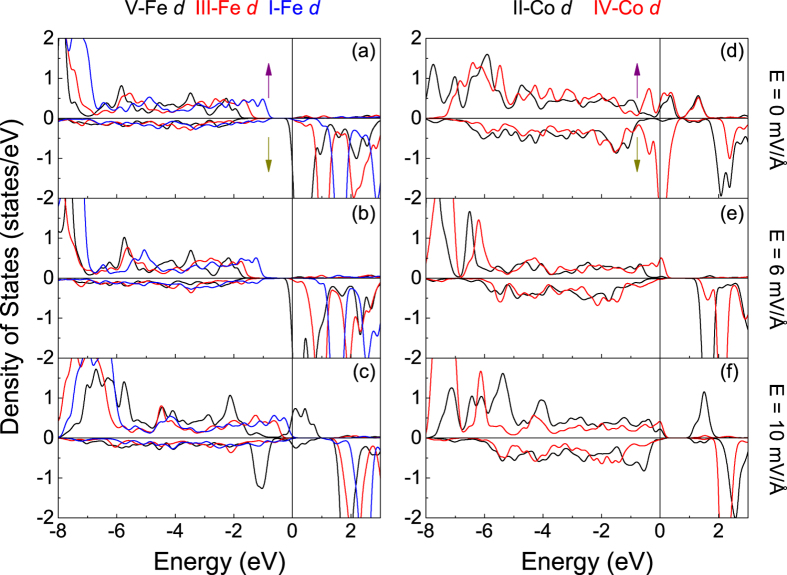
Partial DOS for (**a**–**c**) Fe and (**d**–**f**) Co in each layer of model BiO-FeO_2_ on different values of electric field. *E*_*F *_= 0 eV.

On the other hand, the ferroelectric displacements are not sensitive to external electric field in model BiO-FeO_2_ as shown in Fig. 4(c), and we believe that the different behavior of models CoO_2_-BiO and BiO-FeO_2_ is a result of termination effect. It is also found in model BiO-FeO_2_ that the electric field reverses spin polarization of Fe/Co ions. Figure 8(d–f) indicate that the spin polarization of IV-Co are reversed from −73% to 100% upon *E* = 6 and 10 mV/Å, While V-Fe are reversed from −100% to 53% upon *E* = 10 mV/Å according to Fig. 8(a,c). These results show that electric field can not only reverse the positive and negative of spin polarization, but also reach a considerable value even 100%. In addition, the synthesis technology of oxides has beem improved significantly, such as MBE MOCVD, etc., which can fabricate high-quality epitaxial films and heterostructures. We take the La_0.7_Sr_0.3_MnO_3_/BiFeO_3_ structures as an example. For the growth of La_0.7_Sr_0.3_O terminated La_0.7_Sr_0.3_MnO_3_ film, Yu *et al.* modify the SrTiO_3_ substrate from TiO_2_ termination to SrO termination^62^. To achieve this, a very thin layer (2.5 unit cells) of SrRuO_3_ was grown on SrTiO_3_, or growing one monolayer of SrO on the TiO_2_ terminated SrTiO_3_ substrate. And Kim *et al.* also fabricated the BiFeO_3_-MnO_2_-terminated and BiFeO_3_-(La,Sr)O-terminated La_0.7_Sr_0.3_MnO_3_ structures in similar method^63^. Therefore, the energetically unfavored termination can be achieved by inserting specific monolayer in the stable termination. The prediction of ferroelectric metallic characteristics in BiFeO_3_/BiCoO_3_ bilayers is meaningful for the expertimental research, which can provide opportunities for developing novel functional electronic devices.

## Conclusion

In summary, we investigate the electronic structure of BCO/BFO(001) bilayers with different terminations based on first-principles calculations. The multiferroic insulator BCO and BFO transform into metal in all of three models. Particularly, energetically favored model CoO_2_-BiO exhibits ferroelectric metallic properties and external electric field enhances the ferroelectric displacements markedly. The metallic character is mainly associated to the *e*_*g*_ electrons of Fe/Co ions and these electrons simultaneously hybridize with O *p* electrons around *E*_*F*_, yet the *t*_*2g*_ electrons are responsible for ferroelectric properties. Therefore, the division of *e*_*g*_ and *t*_*2g*_ electrons as well as *e*_*g*_-*p* hybridization provide conditions to the coexistence of ferroelectric and metallic properties. These special behaviors of electrons are influenced by the interfacial electronic reconstruction with formed Bi-O electrovalent bond, which breaks O_A_-Fe/Co-O_B_ coupling partially. Besides, strong interfacial coupling changes the Fe/Co magnetic moments and external electric field reverses spin polarization of Fe/Co ions efficiently, reaching a maximum of 100%. Our results demonstrate that interfacial coupling and electric field play key roles on the novel ferroelectric metallic properties of model CoO_2_-BiO, which provides opportunities for developing functional nanoelectronic devices. We hope that our theoretical prediction on the ferroelectric metallic properties and corresponding electric field effect can stimulate further experimental study.

## Additional Information

**How to cite this article**: Yin, L. *et al.* Ferroelectric Metal in Tetragonal BiCoO_3_/BiFeO_3_ Bilayers and Its Electric Field Effect. *Sci. Rep.* **6**, 20591; doi: 10.1038/srep20591 (2016).

## Figures and Tables

**Table 1:** The average values of Bi-O and B-O polar displacements in one layer along [001] direction in three models within different values of external electric field, B = Co/Fe.

	Bulk	CoO_2_-BiO (mV/Å)			CoO_2_-FeO_2_ (mV/Å)			BiO-FeO_2_ (mV/Å)		
		*E *= 0	*E *= 6	*E *= 10	*E *= 0	*E *= 6	*E *= 10	*E *= 0	*E *= 6	*E *= 10
*d*_Bi-O_	0.803	0.582	0.654	0.646	0.268	0.248	0.273	0.364	0.403	0.365
*d*_B-O_	0.643	0.402	0.485	0.455	0.167	0.147	0.188	0.172	0.227	0.157
*W*_*sep*_	–	11.474	12.378	11.754	−1.998	−3.150	−2.886	6.442	8.900	7.626

Corresponding cohesive energy *Wsep* is listed.

**Table 2:** The calculated magnetic moments (μ_B_) of Fe and Co in each layer of three models as compared to the bulk.

Atom		Bulk	CoO_2_-BiO (mV/Å)			CoO_2_-FeO_2_ (mV/Å)			BiO-FeO_2_ (mV/Å)		
			*E *= 0	*E *= 6	*E *= 10	*E *= 0	*E *= 6	*E *= 10	*E *= 0	*E *= 6	*E *= 10
Co	I	3.035	3.009	2.902	3.046	2.898	2.516	0.889	–	–	–
	II		–	–	–	–	–	–	1.829	3.067	1.809
	III		3.032	3.040	2.808	2.633	2.575	2.696	–	–	–
	IV		–	–	–	–	–	–	1.698	2.972	2.988
	V		2.624	2.642	1.052	2.017	3.016	2.228	–	–	–
Fe	I	4.108	–	–	–	3.936	3.910	3.969	4.120	4.099	4.125
	II		4.076	4.093	4.090	–	–	–	–	–	–
	III		–	–	–	3.624	3.644	3.741	4.084	4.092	4.096
	IV		3.765	3.605	3.573	–	–	–	–	–	–
	V		–	–	–	3.841	2.797	1.695	4.010	3.968	2.861
Total		0.000	0.000	0.000	0.000	0.000	0.000	0.000	0.000	0.000	0.000
